# Low-acuity emergency department use among patients in different primary care models in Hamilton and Ontario

**DOI:** 10.1177/08404704211012027

**Published:** 2021-05-10

**Authors:** Olivia Ly, David Price, Refik Saskin, Michelle Howard

**Affiliations:** 1 Michael G. DeGroote School of Medicine, 12362 McMaster University, Hamilton, Ontario, Canada.; 2 152996 Department of Family Medicine, McMaster University, Hamilton, Ontario, Canada.; 3 50010 ICES, Hamilton, Ontario, Canada.

## Abstract

Jurisdictions such as Hamilton, Ontario, where most primary care practices participate in patient enrolment models with enhanced after-hours access, may demonstrate overall improved health equity outcomes. Non-urgent Emergency Department (ED) use has been suggested as an indicator of primary care access; however, the impact of primary care access on ED use is uncertain and likely varies by patient and contextual factors. This population-based, retrospective study investigated whether or not different primary care models were associated with different rates of non-urgent ED visits in Hamilton, a city with relatively high neighbourhood marginalization, compared to the rest of Ontario from 2014/2015 to 2017/2018. In Ontario, enrolment capitation-based practices had more non-urgent ED visits than non-enrolment fee-for-service practices. In Hamilton, where most of the city’s family physicians are in enrolment capitation-based practices, differences between models were minimal. The influence of primary care reforms may differ depending on how they are distributed within regions.

## Background

Approximately 17% of Emergency Department (ED) visits in Canada are due to non-urgent complaints that would be more appropriately managed in primary care settings. In Ontario, such ED visits may be associated with greater healthcare costs^1^ as well as reduced continuity of care and long wait times for patients with low-acuity needs.^2^

The factors that influence patients to access an ED rather than primary care are varied and not completely explained by access to primary care. Although having a regular family physician is associated with lower ED use for non-urgent needs,^3^ socioeconomic factors such as material and social deprivation,^4^ lower education,^5^ age younger than 65 years,^6^ and rural habitation^7^ are also associated with non-urgent ED use. Features of a primary care practice may also influence ED visitation. Numerous studies have attempted to characterize the impact of key reform elements, including after-hours access,^8^ patient enrolment,^9–11^ roster size,^12^ and remuneration model.^13,14^ However, inconsistent conclusions complicated by interactions with patient factors make it difficult to conclude which models or model features contribute to non-urgent ED use at a patient or practice level. It is likely that at local levels, primary care providers adapt ways of working to the population context beyond their individual practice.^15^ Consequently, examining outcomes related to primary care organization at the jurisdiction level may yield new insights.

The city of Hamilton, Ontario, has a population of approximately 500,000 and is situated in the Greater Toronto Hamilton Area of southern Ontario. Family physicians in Hamilton were among the earliest adopters of primary care reform models in Ontario, including the Family Health Team (FHT) model, which now covers the majority of Hamilton residents.^16,17^ A capitation and multidisciplinary team-based model, FHTs were designed to meet complex patient needs. Provincially, FHT models have been found to roster patients who are healthier and of higher socioeconomic status compared to some other models^18^; however, this is not perceived to be the case in Hamilton due to the broad distribution and coverage of FHTs across the city. Hamilton also has greater socioeconomic disparities due to neighbourhoods with concentrated poverty compared to Ontario overall.^19^

The objective of this study was to characterize primary care patient populations and their non-urgent ED use, as well as to explore the value of conducting region-specific assessments of primary care models. To do this, we described the occurrence of non-urgent ED use by patients’ sociodemographic characteristics and by primary care model in the city of Hamilton compared to the rest of the province from 2014/2015 to 2017/2018 to determine whether patterns observed across Ontario held in Hamilton.

## Methods

### Study design and setting

We conducted a cross-sectional study describing the frequency of low-acuity ED visits by people affiliated with different primary care models in the city of Hamilton, Ontario, and in Ontario overall. Provincial health insurance covers the vast majority of residents and funds most medically necessary hospital and physician services.

### Population and data source

We used population health administrative databases in Ontario held at ICES. ICES is an independent, non-profit research institute funded by an annual grant from the Ontario Ministry of Health (MOH) and the Ministry of Long-Term Care (MLTC). As a prescribed entity under Ontario’s privacy legislation, ICES is authorized to collect and use health care data for the purposes of health system analysis, evaluation and decision support. Secure access to these data is governed by policies and procedures that are approved by the Information and Privacy Commissioner of Ontario.

The databases used were the ICES Physician Database for information on family physicians’ primary care model and location and the Registered Persons Database (RPDB) for patients’ demographic data. Patient postal codes from RPDB are linked to Canadian census data using the Postal Code Conversion File to assign a neighbourhood income quintile to each patient. The Primary Care Population (PCPOP) is an ICES-derived database of patients with a primary care physician and includes patients’ primary care enrolment model and prevalent chronic conditions. For ED data, the National Ambulatory Care Reporting System (NACRS) was used. These datasets were linked using unique encoded identifiers and analysed at ICES.

### Primary care models in Ontario

Ontario houses a diverse array of primary care models (Table 1).^20^ Capitation-based models include Family Health Organizations (FHO) and Family Health Networks. Patients in these models are highly encouraged to be enrolled to a physician, and compensation is predominantly through a “capitated” rate for each rostered patient, which covers a defined basket of core primary care, with additional fees and premiums. Some FHO physicians work in FHTs that include funding for allied health professionals. Otherwise, these capitation-based models are often physician-only practices (referred to in results as CAP).

**Table 1.** Primary care models in Ontario as labelled for study analysis

Model	Remuneration scheme	Solo vs group practice	Patient enrolment	Mandated after-hours care	Funding for inter-professional clinicians
Capitation-based models					
FHT	Capitation with premiums for specific services	Group (3+)	Highly encouraged	✓	✓
CAP	Capitation with premiums for specific services	Group (3+)	Highly encouraged	✓	
Enhanced fee-for-service models					
CCM	Fee-for-service with some capitation payments and premiums for specific services	Solo	Encouraged	✓	
FHG	Fee-for-service with some capitation payments and premiums for specific services	Group (3+)	Encouraged	✓	
Other					
NOG	Fee-for-service	Solo	None		

Abbreviations: CAP, Capitated models that do not include funding for non-physician providers; CCM, Comprehensive Care Model; FHG, Family Health Group; FHT, Family Health Team, which includes funding for non-physician providers; NOG, not otherwise grouped.

Enhanced fee-for-service models include the Comprehensive Care Model (CCM) and Family Health Groups (FHG). Patients are encouraged but not required to be enrolled to a physician, and physicians are paid through fee-for-service billings along with some capitation payments and premiums. Physicians commit to providing comprehensive primary care and after-hours care.

Other physicians not in reformed models (referred to in this article as “not otherwise grouped” [NOG]) include traditional fee-for-service solo physicians, locum physicians, and physicians who work in walk-in clinics. Payment is often exclusively fee-for-service, and there is usually no contractual obligation to provide after-hours care. Patient enrolment is not standard.

Models specific to certain regions such as Northern Ontario were not included because there are no comparators in Hamilton. The Community Health Centre and nurse practitioner-led models could not be examined because the data are not included in the routinely available data holdings used for the study.

### Study cohort

We used data from four fiscal years, 2014/2015 through 2017/2018. We excluded people who were ineligible for provincial health insurance, did not have a valid health card, whose date of death was before April 1, 2014, were living in an institution such as long-term care facility, or who had a non-Ontario postal code on April 1, 2014.

ICES uses an algorithm based on a database created by the MOHLTC when people enroll in a primary care model. For non-enrolled people, the family physician from whom the patient received the highest cost of services over two years is assigned as the family physician (ie, virtual rostering).

### Measurements

We used the Canadian Triage and Acuity Scale level 4 (less urgent) or 5 (non-urgent) from the NACRS data as the definition of a “low-acuity” ED visit, which in this report is used interchangeably with “non-urgent.”

We categorized patients according to sex and age. As an indicator of socioeconomic status, we used the Ontario marginalization index (ON-Marg). The ON-Marg is a census-based index developed to quantify the degree of marginalization occurring across the province of Ontario.^21^ The scores were divided into quintiles, whereby quintile 1 corresponded to living in the least marginalized (ie, most privileged) neighbourhood and quintile 5 corresponded to living in the most marginalized neighbourhood. We examined the distribution of health conditions using the Charlson comorbidity score (0, 1, 2, 3, or more) and included acute myocardial infarction,^22^ asthma,^23,24^ congestive heart failure,^25^ diabetes,^26^ and hypertension^27^ based on previously validated case ascertainment definitions. The presence of a mental health issue was defined as an encounter related to mental health in the last two years.

### Analysis methods

After descriptive analysis of patient characteristics and low-acuity ED visits by primary care model for each year yielded nearly identical results, we combined the data across the four years summatively. The outcome of interest was the occurrence of any low-acuity ED visit. We calculated the distributions of patient characteristics within each primary care model overall and the distributions among the subset who had at least one low-acuity ED visit.

### Research ethics

The use of data in this project was authorized under section 45 of Ontario’s Personal Health Information Protection Act, which does not require review by a Research Ethics Board.

## Results

### Primary care model coverage in Hamilton versus Ontario

In Hamilton, the majority of the population receives care from an enrolment-based primary care model (80%; 64% FHT + 16% CAP), whereas across Ontario CAP (31%) and FHT (26%) models provide care to approximately half of the population (Table 2).

**Table 2.** Baseline patient characteristics in Hamilton and Ontario by primary care model from 2014/15 to 2017/18

Demographics		Hamilton					Ontario (excluding Hamilton)					
		CAP	FHT	CCM	FHG	NOG	CAP	FHT	CCM	FHG	NOG
N		289,499	1,167,932	47,055	228,835	98,839	15,023,633	12,494,640	1,897,137	15,003,760	3,988,600
Population served (%)		16	64	3	12	5	31	26	4	31	8
Sex (%)	Female	53	52	53	49	49	53	53	51	52	48
	Male	47	48	47	51	51	48	47	49	48	52
Age (%)	0	21	20	22	21	24	18	20	18	20	36
	19-44	32	32	38	35	40	33	32	35	37	31
	45-75	39	40	34	38	32	41	40	39	37	27
	75+	8	9	6	7	4	8	9	7	6	5
Deprivation quintile (%)	1	31	20	22	22	14	26	22	19	21	21
	2	22	18	18	17	14	22	22	20	21	19
	3	15	18	16	16	16	19	20	20	19	18
	4	15	20	19	18	21	17	19	20	19	18
	5	16	25	25	28	34	16	17	22	20	22
	Missing	0	0	0	0	1	0	1	1	0	1
Chronic condition (%)	AMI	2	2	1	1	1	1	2	1	1	1
	Asthma	13	12	15	13	13	15	15	15	15	16
	CHF	2	2	2	2	1	2	2	2	1	1
	COPD	6	7	6	8	4	7	8	7	5	5
	DM	9	10	9	11	7	11	10	11	11	7
	HTN	22	23	19	11	18	24	23	23	22	16
	MH	21	19	21	26	20	20	19	22	21	20

Abbreviations: AMI, acute myocardial infarction; CAP, Capitated models that do not include funding for non-physician providers; CCM, Comprehensive Care Model; CHF, congestive heart failure; COPD, chronic obstructive pulmonary disease; DM, diabetes mellitus; FHG, Family Health Group; FHT, Family Health Team; HTN, hypertension; MH, mental health; N, the sum of all patients from each annual dataset; NOG, not otherwise grouped.

### Patient population characteristics by primary care model in Hamilton versus Ontario

There were greater proportions of people in age categories below 45 years in Hamilton compared to the rest of Ontario. In terms of patient population characteristics in the different models, in both Hamilton and Ontario, the CAP and FHT models had the highest proportions aged 75 and over. In Hamilton, there tended to be a lower prevalence of chronic obstructive pulmonary disease, diabetes, and hypertension compared to Ontario, regardless of primary care model, which may be related to the slightly younger age distribution in Hamilton.

In Hamilton, the NOG model served the highest proportion of patients in the most marginalized ON-Marg quintile. In both Hamilton and Ontario, the CAP model served the highest proportion of patients in the least marginalized ON-Marg quintile.

### Low ED-use patient population characteristics by primary care model in Hamilton versus Ontario

In Hamilton, there was less variability in the proportion of patients having a non-urgent ED encounter between the primary care models compared to the variability by model in the rest of Ontario (Table 3). In Hamilton, the proportions in different models were similar (ranging from 10.2% to 11.4%). In the rest of Ontario, the range was greater. The proportion of patients with non-urgent ED use in FHT practices was almost double that of FHG practices (14.0% vs 7.6%).

**Table 3.** Characteristics of patients in Hamilton and Ontario accessing emergency department services for low-acuity presentations by primary care model from 2014/2015 to 2017/2018

Demographics		Hamilton					Ontario (excluding Hamilton)				
		CAP	FHT	CCM	FHG	NOG	CAP	FHT	CCM	FHG	NOG
Total number of low-acuity visits		38,089^a^	164,216	6,341	31,161	15,553	1,919,030	2,623,093	253,534	1,484,647	611,156
% of patients with at least one low-acuity ED visit		10.2	11.0	10.4	10.2	11.4	9.5	14.0	9.6	7.6	10.6
Age (%)	0	31	30	32	28	33	23	24	22	24	35
	19-44	33	33	36	36	40	25	33	36	38	33
	45-75	30	30	27	31	25	34	34	34	32	27
	75+	7	7	6	5	3	9	10	8	6	5
Deprivation quintile (%)	1	24	16	19	17	10	19	14	14	18	16
	2	21	17	17	14	13	21	19	18	20	16
	3	16	17	18	15	16	20	20	20	19	17
	4	17	21	20	19	20	19	22	21	20	19
	5	21	28	26	35	36	20	22	26	23	27
	Missing	1	1	0	1	5	1	2	2	1	5
Gradient of quintile 5 to 1 (%)^b^		7	5	3	6	5	6	14	6	3	8
Chronic condition (%)	AMI	2	2	1	1	1	2	2	2	1	1
	Asthma	17	16	20	19	16	20	19	21	21	20
	CHF	2	2	2	2	1	3	3	3	2	2
	COPD	7	7	7	9	4	9	11	10	8	8
	DM	9	10	9	11	7	12	13	13	11	11
	HTN	19	19	17	20	15	24	24	24	22	18
	MH	26	24	27	35	26	27	25	32	20	29

Abbreviations: AMI, acute myocardial infarction; CAP, Capitated models that do not include funding for non-physician providers; CCM, Comprehensive Care Model; CHF, congestive heart failure; COPD, chronic obstructive pulmonary disease; CTAS, Canadian Triage and Acuity Scale; DM, diabetes mellitus; ED, emergency department; FHG, Family Health Group; FHT, Family Health Team; HTN, hypertension; MH, mental health; NOG, not otherwise grouped; ON-Marg, Ontario marginalization.

^a^ Low-acuity visits are defined as having a CTAS score ≥4.

^b^ The gradient was calculated as the difference between the proportion of patients with a low-acuity ED visit in ON-Marg quintile 1 and quintile 5. The proportion of patients with such a visit is the total number of low-acuity ED users in a quintile divided by the total number of patients in that quintile. A gradient of 7% indicates that those in the most marginalized quintile experience a 7% greater absolute risk of an ED encounter compared to those in the least marginalized quintile.

The proportion of patients with chronic conditions who had a low-acuity ED encounter followed a similar pattern to the distribution of chronic disease within the overall cohort for that model. However, there were higher proportions of patients with asthma and mental health and lower proportions with hypertension among low-acuity ED attenders across all models in both Hamilton and Ontario.

All primary care models demonstrated a socioeconomic (ON-Marg) gradient for the proportion of patients with a low-acuity ED encounter. Those experiencing more marginalization were overrepresented among low-acuity ED visits, and those experiencing less marginalization were underrepresented. However, there were notable differences between primary care models, more so for Ontario compared to Hamilton.

The gradient calculation in Table 3 shows the difference between the deprivation quintile gradient among the population who had a non-urgent ED visit in that primary care model, to the deprivation quintile gradient for all patients in the primary care model. A difference of 0% would indicate no difference in the deprivation quintile gradient in the non-urgent ED users compared to the full population. There was a difference for all primary care models, suggesting that people with lower socioeconomic status were overrepresented among non-urgent ED users. The range of differences between primary care models was greater for Ontario (range between 3% and 14%) compared to the range for Hamilton (range between 3% and 7%). The FHT model for Ontario exhibited the greatest difference in deprivation quintile gradient between the full practice population and the subgroup of non-urgent ED users (difference in gradients 14%). This difference was 5% in Hamilton.

## Discussion

We described low-acuity ED encounters between 2014/2015 and 2017/2018 in Hamilton compared to the rest of the province of Ontario. In both city and province, the NOG model demonstrated rates of low-acuity ED use that were only slightly higher than the average, despite serving a greater proportion of young and highly marginalized people and not having any of the key features of reformed models. The FHG practices in both Hamilton and Ontario had the lowest rates of low-acuity ED encounters with both a higher- and normal-risk patient population, respectively. Both NOG and FHG models are predominantly fee-for-service, which is consistent with a study by Glazier et al. that found enhanced fee-for-service practices were associated with more after-hours service and fewer ED encounters.^14^ Fee-for-service remuneration may encourage more frequent visits and ultimately enhance access to care.^12,28^

Additionally, important differences were observed between municipal and provincial data that could not have been predicted by model and demographic data alone. The proportion of patients with a low-acuity ED visit in Hamilton FHTs was notably lower than in Ontario’s FHTs, and the marginalization gradient in ED encounter was among the lowest within Hamilton FHTs but was particularly steep for Ontario. These trends occurred counterintuitively in the context of Hamilton FHTs serving a greater proportion of highly marginalized patients with an otherwise similar age and morbidity distribution compared to Ontario. We hypothesize that these findings may be due to the community distribution of care models. Currently, access to team-based primary care in Ontario is less available in the major urban centres and northern communities where the need for primary care is the greatest.^29^ Our data suggest that the widespread coverage of FHTs in Hamilton may expand its reach to the low-income, complex patients which may help mitigate health disparities. Combined with earlier conclusions that fee-for-service-based models may also reduce non-urgent ED use, our findings support a recent call to make enrolment, team-based care with several options for remuneration the standard form of care for the majority of Ontarians.^30^

This study had several limitations. We did not attempt to quantify the risks of low-acuity ED use by patient characteristics nor to conduct regression analyses to control for the potential confounding effects of patient factors on the probability of low-acuity ED use. Risk factors for low-acuity ED use have been previously described in the literature, and we aimed to obtain a population-level comparison of low-acuity ED use across primary care models. Additionally, Ontario is a large province with many heterogeneous communities. Although a comparison of low-acuity ED use in Hamilton, a mid-sized urban centre, to the rest of Ontario yields an initial understanding of ED visitation trends among different primary care models in Hamilton, further comparison to other mid-sized cities in Ontario and across Canada may produce additional insights.

In Hamilton, a city with a comparatively large gradient of marginalization and a majority of family physicians working in enrolment capitation-based models with interprofessional teams, the differences in low-acuity ED use between primary care models were attenuated compared to the rest of Ontario, while having overall low-acuity ED use that is comparable to the rest of Ontario. The influence of primary care reforms may differ depending on how they are distributed in regions and unique local features. Future research should explore the community-level impacts of interprofessional primary care teams and how the distribution of primary care models within a community impacts healthcare utilization across the system.
